# Pathological survey on *Temnodontosaurus* from the Early Jurassic of southern Germany

**DOI:** 10.1371/journal.pone.0204951

**Published:** 2018-10-24

**Authors:** Judith M. Pardo-Pérez, Benjamin P. Kear, Heinrich Mallison, Marcelo Gómez, Manuel Moroni, Erin E. Maxwell

**Affiliations:** 1 Staatliches Museum für Naturkunde Stuttgart, Stuttgart, Germany; 2 Vicerrectoría de Investigación y Postgrado, Universidad de Magallanes, Punta Arenas, Chile; 3 Museum of Evolution, Uppsala University, Uppsala, Sweden; 4 Museum für Naturkunde Berlin, Berlin, Germany; 5 Escuela de Medicina Veterinaria, Facultad de Ciencias Veterinarias, Universidad Austral de Chile, Campus Isla Teja, Valdivia, Chile; State Museum of Natural History, GERMANY

## Abstract

Paleopathologies document skeletal damage in extinct organisms and can be used to infer the causes of injury, as well as aspects of related biology, ecology and behavior. To date, few studies have been undertaken on Jurassic marine reptiles, while ichthyosaur pathologies in particular have never been systematically evaluated. Here we survey 41 specimens of the apex predator ichthyosaur *Temnodontosaurus* from the Early Jurassic of southern Germany in order to document the range and absolute frequency of pathologies observed in this taxon as a function of the number of specimens examined. According to our analysis, most observed pathologies in *Temnodontosaurus* are force-induced traumas with signs of healing, possibly inflicted during aggressive interactions with conspecifics. When the material is preserved, broken ribs are correlated in most of the cases with traumas elsewhere in the skeleton such as cranial injuries. The range of cranial pathologies in *Temnodontosaurus* is similar to those reported for extinct cetaceans and mosasaurs, which were interpreted as traces of aggressive encounters. Nevertheless, *Temnodontosaurus* differs from these other marine amniotes in the absence of pathologies in the vertebral column, consistent with the pattern previously documented in ichthyosaurs. We did not detect any instances of avascular necrosis in *Temnodontosaurus* from southern Germany, which may reflect a shallow diving life style. This study is intended to provide baseline data for the various types of observed pathologies in large ichthyosaurs occupying the ‘apex predator’ niche, and potentially clarifies aspects of species-specific behavior relative to other ichthyosaurs and marine amniotes.

## Introduction

Ichthyosaurs a group of highly specialized Mesozoic were marine reptiles that ranged from the Early Triassic to the early Late Cretaceous (250–90 Ma) [1]. Large-bodied macrophagous forms appeared early in the evolutionary history of the group (Middle Triassic: [2], and ichthyosaurs continued to occupy apex predator niches until the end of the Early Jurassic [3]; in later time periods this niche was occupied by large pliosaurids. *Temnodontosaurus* is a classic example of a large-bodied predatory ichthyosaur from the Early Jurassic [1]. The genus included some macrophagous species [4], characterized by skulls and jaws over 1 m in length, with the largest being over 1.9 m long [5]. The total body length of the largest *Temnodontosaurus* is estimated at up to 15 m (reviewed by [5]). Gastric contents indicate predation on marine reptiles, including other ichthyosaurs and cephalopods [6].

Paleopathologies document skeletal damage in extinct organisms and can be used to infer aspects of ecology and behavior [7,8]. To date, few detailed studies of palaeopathologies have been undertaken on Jurassic marine reptiles, and ichthyosaur pathologies in particular have never been systematically evaluated at a genus level. Anecdotal reports include examples of broken and healed ribs, articular disease, and ankylosis (reviewed by [9]). Broken ribs with pseudarthroses are the most frequently reported pathology in *Temnodontosaurus*, specifically, but are known from only two cases: a specimen of the Toarcian species *T*. *trigonodon*, and another from the Pliensbachian species *T*. *nuertingensis* [10]. A single instance of avascular necrosis has also been reported in an isolated femur referred to *Temnodontosaurus* sp. [11]). However, personal observation suggests a wider range of pathologies in *Temnodontosaurus* than reflected by the current literature. The broad range of pathological bone modifications observed in *Temnodontosaurus* make this genus a valuable case study to categorize the range and types of pathologies observed in ichthyosaurs, and to establish criteria for differentiating pathologies from other marks on the skeleton.

Here, we survey Early Jurassic specimens of *Temnodontosaurus* from southern Germany in order to document the types of pathologies observed in the genus, and to provide an atlas of paleopathologies in large-bodied ichthyosaurian taxa. This study will provide a baseline of types and frequencies of pathologies in secondarily aquatic marine reptiles occupying the apex predator niche, and large ichthyosaurs more generally, and may clarify some aspects of the paleobiology of these animals, including social behavior.

### Institutional abbreviations

SMNS: Staatliches Museum für Naturkunde Stuttgart, Germany. GPIT: Geologisches und Paläontologisches Museum Tübingen, Germany. U-MO: Urwelt-Museum Oberfranken, Bayreuth, Germany. UMH: Urwelt Museum Hauff, Holzmaden, Germany.

## Materials and methods

We surveyed 39 specimens of *Temnodontosaurus* from six collections (S1 Table), spanning the Pliensbachian to Toarcian of southwestern Germany. Each specimen was macroscopically examined in detail using a magnifying lens. We measured the total length of premaxilla, dentary, humerus, and femur to obtain an approximate size estimate for individuals and indirectly control for ontogenetic effects. We also recorded the state of preservation and preparation for each specimen. In the cases where pathologies where observed, we documented and described them according to it appearance, texture, dimensions and anatomical location.

**Fig 1:**
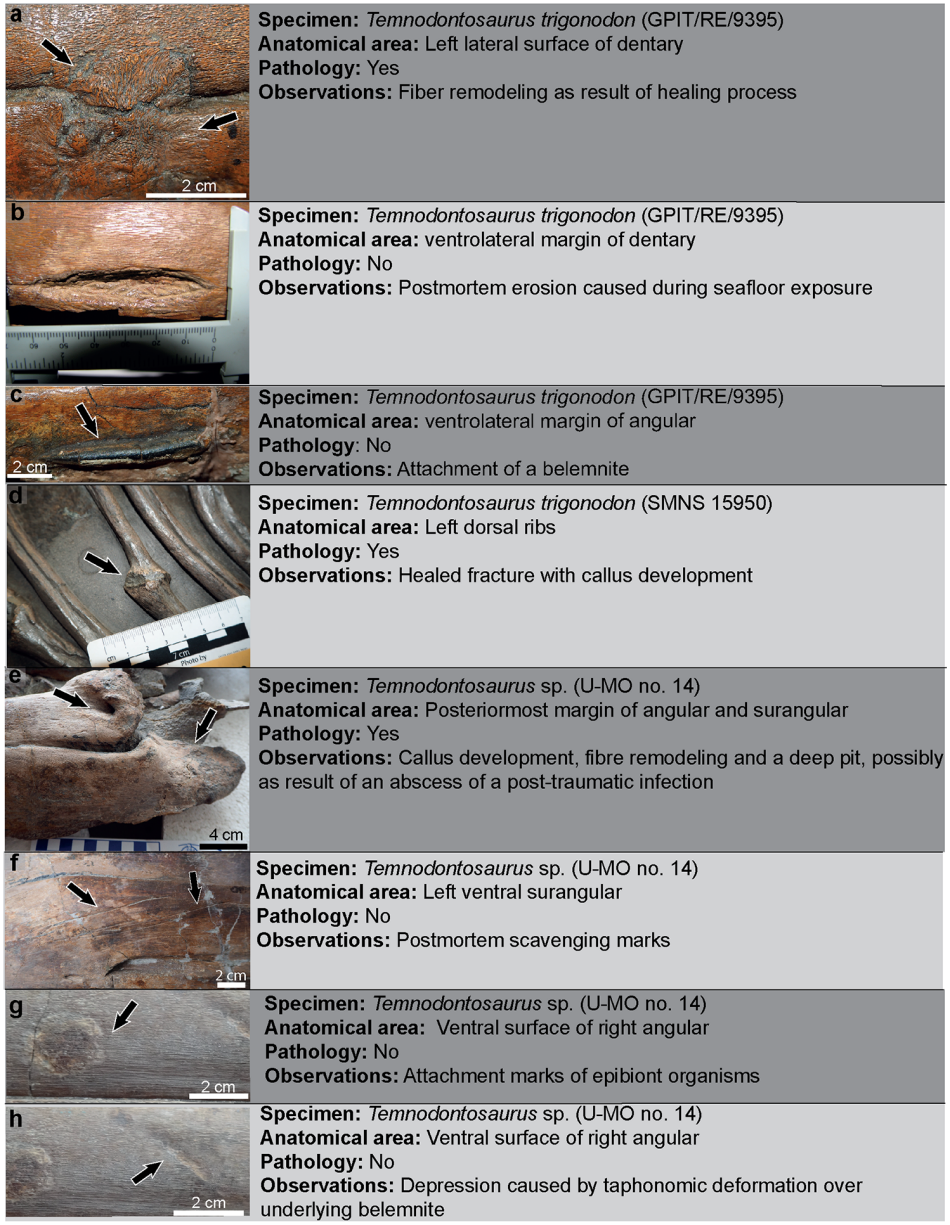
Examples of pathological vs non-pathological bone modifications in *Temnodontosaurus*.

We differentiate pathological skeletal modifications from peri- or postmortem alterations of the bone, including bones fractures that lack signs of healing, signs of postmortem scavenging, attachment traces left by encrusting epibionts, erosion during sea floor exposure, compression or plastic deformation during taphonomy, and damage incurred during excavation and/or preparation (Fig 1b, 1c, 1f, 1g and 1h). We identified pathological specimens by the presence of callus formation or bone fibre remodeling (Fig 1a, 1d and 1e), developed as consequence of a trauma or a post-traumatic disease. Our approach therefore identifies only the minimum possible number of pathological individuals: all cases with skeletal abnormalities lacking these characteristics are not considered in this study.

**Fig 2:**
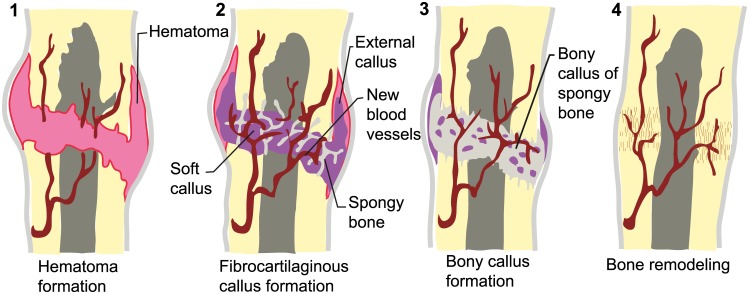
Healing process of bone. Modified from Cummings (2004). **1**. Hematoma formation. **2**. Fibrocartilaginous callus formation. **3**. Bony callus formation. **4**. Bone remodeling.

Bone is a dynamic tissue, continuously deposited and reabsorbed in response to mechanical changes caused by stress or healing of fractures. Osseous remodeling consists of the resorption of a determinate amount of bone performed by osteoclast cells, and the formation of the osteoid matrix by the osteoblasts and later mineralization [12]. This phenomenon occurs in small areas of the cortex or the trabecular surface. The remodeling process can last several days, months or even years, and is expressed at different stages (Fig 2): (1) Reaction: hematoma and inflammation of the affected area by the vessels and tendon rupture plus granulation tissue formation. (2) Fibrocartilaginous callus (reparation): hyaline cartilage and fibrous deposition in the clot forming a fibrocartilaginous soft callus. (3) Endochondral ossification (reparation): Lamellar bone forms a hard callus with woven bone. (4) Remodeling: remodeling to the original contour. In this final stage the correct healing process can fail, leading to the development of a pseudarthrosis ([9], Fig 2d and 2e). In fossils, we can only recognize stages three and four. Post-mortem alterations and perimortem injuries are clearly differentiated from pathological bones because of the absence of healing represented by fibre remodeling and callus formation.

For analytical purposes we classified the pathologies in our *Temnodontosaurus* sample following the scheme outlined in [9].

### Trauma with evidence of healing

Fiber remodeling of bone as a consequence of a wound produced by sudden physical injury (e.g. from violence or accident). Evidence of callus development (tissue overgrowth) around the damaged area may also be present (Fig 1a). Infection may also occasionally occur and result in abscess formation (Fig 1e).

### Articular disease

**Fig 3:**
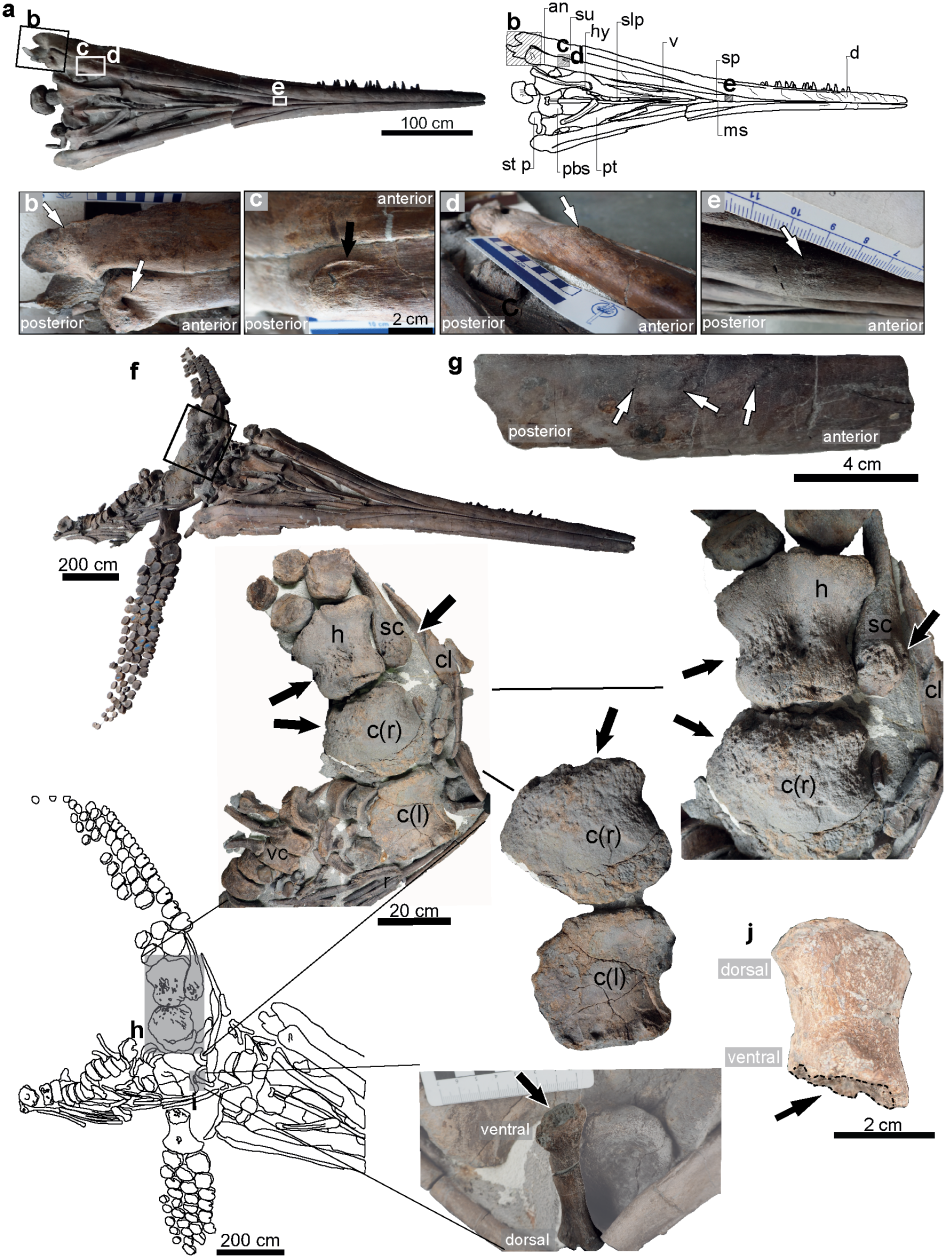
*Temnodontosaurus* sp., Jurensismergel Formation (upper Toarcian). Urwelt-Museum Oberfranken, Bayreuth, Germany; Rabold and Eggmaier (2013) specimen no. 14. **a**. Overview of the skull exposed in ventral view, indicating the pathological areas in b, c, d and e. **b**. Posteriormost end of the right angular and surangular showing deformed, rugose tissue on the surangular and a deep pit in the angular. **c**. Long groove on the right ventrolateral margin of the right surangular. The margins are made of a crest of reactive bone. **d**. The same reactive bone crest in left ventrolateral view. **e**. Small protuberance of reactive bone on the ventral surface of the right splenial. **f**. Complete preserved skeleton in ventral view indicating the pathological portion of the right pectoral girdle. **g**. A broken fragment of premaxilla indicating the three areas with fibre remodeling with the white arrows. **h**. The pathological right coracoid, scapula, and humerus in ventral view, showing the pitting and the dorsoventral thickening compared with the elements of the left pectoral girdle. **i-j**. Two broken anterior dorsal ribs showing a rugose concavity at the distal end, which represents the proximal callus of a pseudarthrosis. Abbreviations: **an**, angular; **d**, dentary; **hy**, hyoid; **ms**, mandibular symphysis; **pb**, parabasisphenoid; **pt**, pterygoid; **slp**, sclerotic plates; **sp**, splenial; **st p**, stapes; **su**, surangular; **v**, vomer.

Originates as the result of inflammation or an interruption of the blood supply to the bone and cartilage of an articular surface causing damage to the joint. Articular disease can be generated by trauma, genetic disorders, or joint fatigue as result of certain movements. Osteoarthritis and avascular necrosis are examples of this type of disease (see [9], Fig 3).

### Ankylosis

Trauma or disease causing partial or total postnatal fusion of normally separate skeletal elements at joint spaces.

To compare our observations obtained from the *Temnodontosaurus* sample with data compiled from the literature [9], we categorized the skeleton of each specimen into six anatomical units, modified from [13]: (1) skull, (2) anterior (cervical+dorsal) vertebral column, (3) posterior (sacral+caudal) vertebral column, (4) thoracic ribs, (5) pectoral girdle and forefin, (6) pelvic girdle and hind fin. We compared the relative frequency of pathologies per anatomical unit, in order to obtain an estimate of frequency of pathologies relative to the number of individuals preserved, as well as the type of pathology most represented in *Temnodontosaurus*. Extremely fragmentary material consisting of isolated elements (e.g., single ribs, a vertebral centra, jaw fragments) were excluded to avoid statistical bias.

## Results

Most of the specimens from the collections are strongly taphonomically compressed. They are typically prepared as flat panel mounts with large sections of the skeleton still embedded in matrix. The only exception are remains recovered from the Jurensismergel Formation, housed at the Urwelt-Museum Oberfranken, which are preserved in three dimensions. We provide a detailed description of the three-dimensionally preserved and recently collected and prepared U-MO no. 14 [14], to illustrate the range of pathologies observed as well as post-mortem artifacts. We also provide information on other pathological specimens, focusing only on unambiguous pathological structures.

### Detailed description of pathological specimens

#### *Temnodontosaurus* sp. (U-MO Rabold and Eggmaier (2013) no. 14). Jurensismergel Formation (upper Toarcian), Mistelgau, Germany

An incomplete skeleton prepared in ventral view, preserving the skull, anterior vertebral column, ribs and the right and left pectoral girdle and forelimbs. This specimen is inconsistent with the early-middle Toarcian species *Temnodontosaurus trigonodon*; taxonomic work is ongoing.

Potentially pathological structures were observed in the skull, ribs, and the right pectoral girdle (Fig 3).

#### Skull

A broken fragment of the anterior right premaxilla shows three marks along its dorsal edge characterized by bone fibre remodeling (Fig 3g). In the lower jaw, the posterior edge of the right angular has been replaced by a thickened and rugose region, possibly resulting from callus formation; immediately anterior to this thickened section a deep pit oriented anteroposteriorly continues under the thickened area (Fig 3b). A long groove runs longitudinally along the ventrolateral margin of the right angular, 10 cm anterior to the pit (Fig 3c). The groove shows a sharp crest of reactive bone at its ventral surface (Fig 3d). Lastly, a small protuberance of reactive bone is observed on the ventral margin of the right splenial, 2.4 cm anterior to the posterior-most end of the mandibular symphysis (Fig 3e). It is of rugose texture and shows fiber remodeling.

#### Ribs

Two partial anterior left dorsal ribs, preserved disarticulated, show an expanded distal end terminating in a rugose concavity (Fig 3i and 3j). This concavity represents callus development formed during pseudarthrosis formation, with the portion of the rib distal to the pseudarthrosis having been taphonomically lost. The pathology is located at 6.1 cm and 3.3 cm from the proximal head of the ribs of the Fig 3i and 3j respectively.

#### Pectoral girdle and forelimb

Pathologies are observed affecting the right coracoid, scapula and humerus (Fig 3f). The right coracoid is wider in all dimensions than the left and has a rugose ventral surface with pitting on its lateral and posteroventral margin. Relative to the left coracoid, the right coracoid is dorsoventrally thickened, and its ventral surface is convex rather than saddle-shaped. Two deep but small notches are observed at the lateral edge where it articulates with the humerus.

The proximal articular surface of the right scapula is thickened and also of rugose texture with several small depressions.

The proximal end of the right humerus is greatly enlarged relative to the left along the anteroposterior axis (Table 1). The surface texture has small pits and rugosities, most noticeable from the mid-diaphysis to the proximal end.

**Table 1:** Measurements (in mm) of right and left humeri of U-MO specimen no. 14 (*Temnodontosaurus* sp.).

	Right humerus	Left humerus
Proximo-distal length	147	147
Proximal width	117	82
Distal width	150	141
Diaphysis width	113	80

#### Non-pathological bone modifications

During our survey we also recognized non-pathological modifications in U-MO no. 14. The modifications in the bones were classified as being caused postmortem, and lacked evidence of healing in the modified area (i.e., callus or osseous fibre remodeling). Three types of modifications were observed: attachment of epibionts and scavenging marks caused during seafloor exposure, and taphonomic deformation, caused after burial.

- Attachment of epibionts: On the ventral surface of the right angular is a circular area eroded around the periphery and discolored in the center (Fig 1g). This is attributed to an attached epibiont, probably a bivalve.
- Scavenging marks: Four scratches were observed on the ventral surface of the left surangular. The marks are thin and shallow, partially filled with sediment, and run roughly parallel to each other and to the long axis of the jaw (Fig 1f). Postmortem scavenging by sharks is a possible cause [15].
- An elongate depression on the ventral surface of the right angular (Fig 1h) was caused by taphonomic deformation over an underlying belemnite (Eggmaier pers. comm.).

### Pathologies observed in other *Temnodontosaurus* specimens

#### *Temnodontosaurus nuertingensis* (SMNS 13488). Numismalismergel Formation (lower Pliensbachian), Nürtingen, Germany

**Fig 4:**
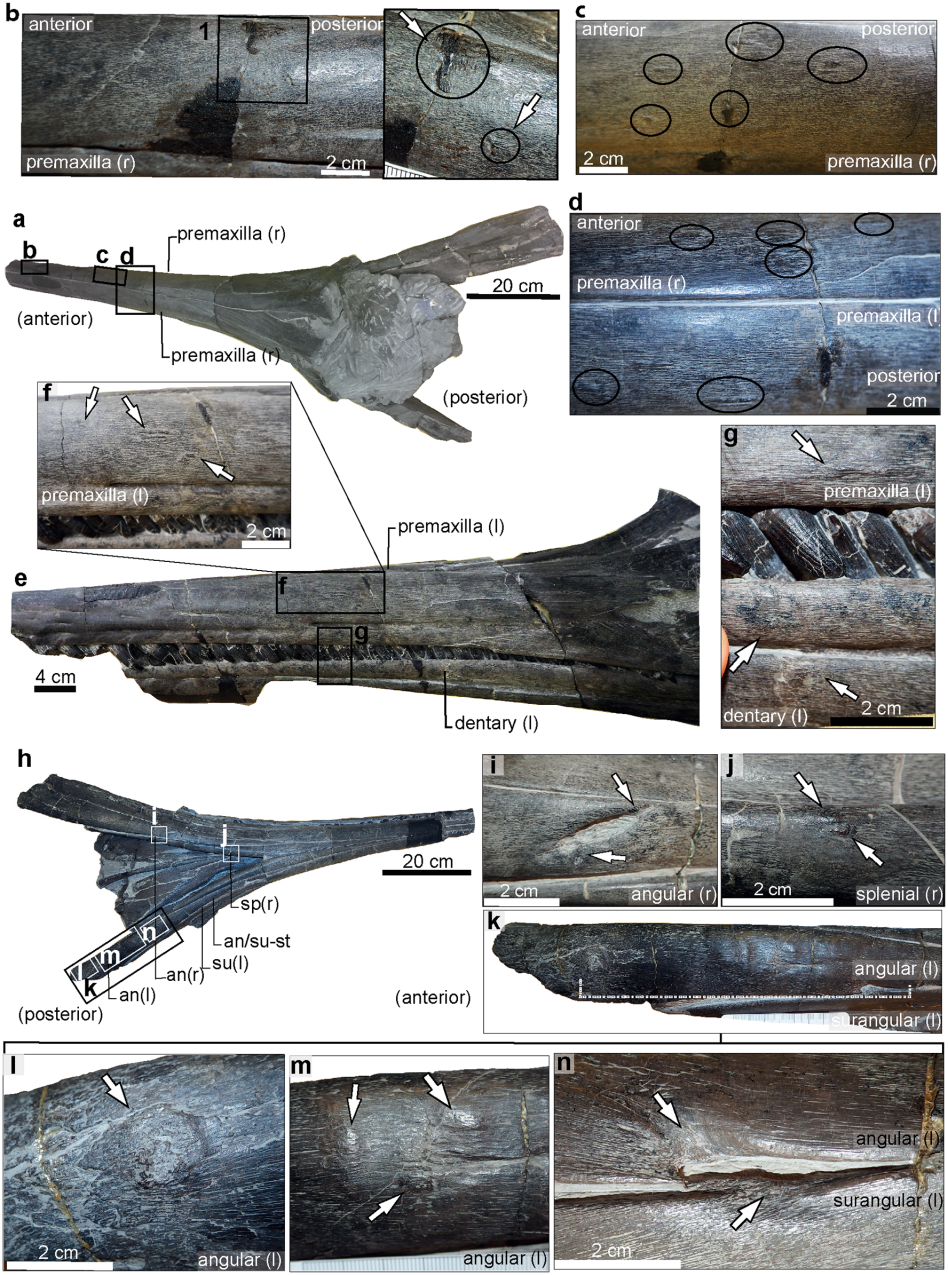
*Temnodontosaurus nuertingensis* (SMNS 13488). Numismalismergel Formation (lower Pliensbachian). **a**. Skull in dorsal view indicating the pathologies in the premaxilla in b, c and d. **b**. Right premaxilla showing two areas of fibre remodeling (inset: magnified view). **c**. Lateral surface of the right premaxilla indicating five small areas of fibre remodeling. **d**. Dorsal view of the right and left premaxillae, indicating areas with fibre remodeling. **e**. Skull in left lateral view indicating pathological areas on the left premaxilla (f) and left dentary (g). **f**. Three small areas with fibre remodeling on the left premaxilla. **g**. Fibre remodeling on the ventrolateral margin of the left premaxilla and the lateral surface of the left dentary. **h**. Skull in ventral view, showing the location of the pathologies illustrated in i-n. **i**. A posteroventral to anterodorsally oriented concavity in the right angular. The arrows indicate slight fibre remodeling at the ends. **j**. A rugose protuberance with fibre remodeling on the right splenial. **k**. Pathological area between the left angular and surangular demarcated by dotted lines. **l**. A small area with callus development on the left angular. **m**. Three small protuberances with fibre remodeling on the lateral margin of the left angular. **n**. A teardrop concavity between the left angular and left surangular with fibre remodeling at its dorsal and ventral corners.

The specimen now consists of a partial skull preserved in three dimensions (Fig 4, S1, S3 and S4 Files; instructions for opening 3D data are found in S6 File), but originally also comprised eight vertebrae, a coracoid, and two proximal ribs with distal pseudarthrosis and callus formation [10,16].

#### Skull

Six small areas with fibre remodeling separated by a few centimeters are present on the lateral surface of the right premaxilla, and four on the left premaxilla from 21.6 cm to 41.5 cm anterior to the external narial opening (Fig 4a, 4e and 4f and S3 File). In addition, multiple mandibular pathologies are observed (Fig 4h). Two small areas with fibre remodeling are observed on the lateral surface of the left dentary at the level of the pathologies observed in the left premaxilla (Fig 4e and 4g). A teardrop-shaped embayment 1.4 cm in length and 1.7 cm high is observed in the dorsalmost left angular along the angular-surangular suture at 28.6 cm from the posterior end of the mandibular symphysis (Fig 4h and 4n and S4 File). The embayment shows fibre remodeling at its posterior edge; fibre remodeling is also present on the ventralmost surangular. Posterior to this embayment, three lesions of reactive bone lie along the ventral angular (total area: 10 cm length and 3.2 cm height) (Fig 4h and 4m). Another lesion of reactive bone with callus formation lies slightly anterior to the posteriormost angular (15.5 cm from the embayment pathology and at 46.2 cm from the posterior end of the mandibular symphysis) (Fig 4h and 4l). On the ventral surface of the right mandible, a rugose protuberance with fibre remodeling (0.6 cm length; 0.4 cm high) is observed on the right splenial (Fig 4h and 4j). The protuberance is observed 47.5 cm from the mandibular symphysis. A diagonal concavity, dorsoventrally orientated, is observed in the right angular (3.2 cm length; 0.5 cm height) at 4.1 cm posterior to the pathology of the right splenial (Fig 4h and 4i). The pathology shows slight fibre remodeling along its edges. It lacks reactive bone deposition, however this may have been removed by aggressive mechanical preparation.

#### Ribs

Two broken ribs with signs of callus development and pseudarthrosis formation were described by [10] in *T*. *nuertingensis*. This material has since been lost.

#### *Temnodontosaurus trigonodon* (SMNS 15950). Posidonienschiefer Formation (lower–middle Toarcian); Holzmaden, Germany

**Fig 5:**
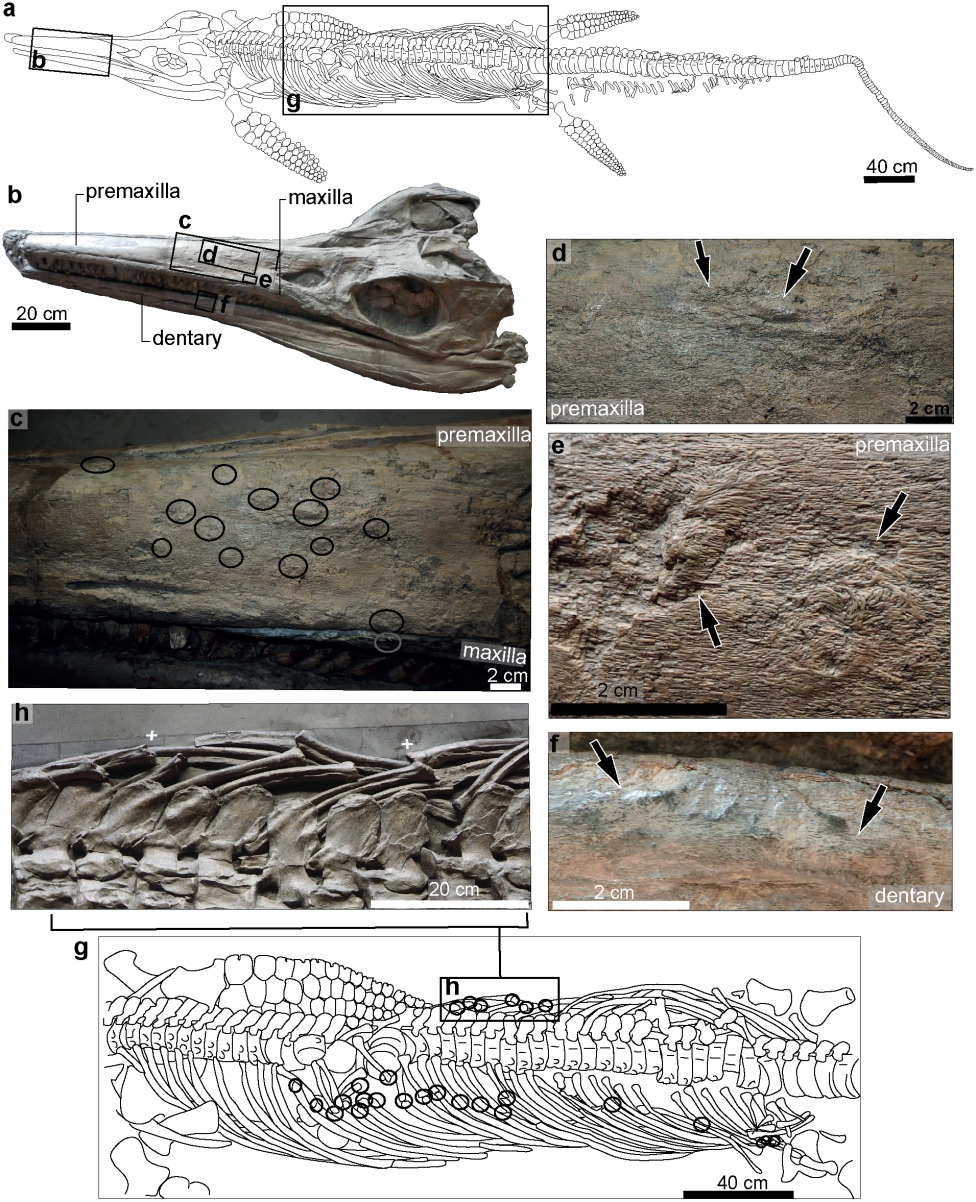
*Temnodontosaurus trigonodon* (SMNS 15950). Posidonienschiefer Formation (lower–middle Toarcian). **a**. Complete skeleton prepared in left lateral view indicating the pathological areas in the skull (b) and ribs (g). **b**. Skull, indicating the detected pathologies of the left premaxilla, maxilla and dentary in c, d, e and f. **c**. Detail of the fibre remodeling observed in the premaxilla indicated in the black circles. **d, e**. Arrows indicating remodeled areas on the lateral surface of the premaxilla. **f**. Left dentary indicating two areas with fibre remodeling on the anteroventral edge. g. left lateral view of the left and right (h) dorsal ribs indicating the broken and healed ribs with the black circles. **h**. Detail of the broken and healed right dorsal ribs.

A complete and articulated specimen, exposed in left lateral view (Fig 5a and S2 File). The specimen is displayed as a slab mount, and severe taphonomic compression has occurred. Mechanical preparation of this specimen was very aggressive; however the pathologies can be clearly distinguished from the damage of the bone caused during preparation. Improperly healed ribs bearing pseudarthroses were previously described in this specimen [10]; however previously undescribed cranial injuries are also present (S5 File).

#### Skull

Twelve roughly circular areas separated by a few centimeters (total area: 28 cm length and 12 cm height) show bone with woven, disoriented fibres and occasional reactive bone deposition on the dorsal and lateral left premaxilla. One roughly circular area with fibre remodeling is in the left maxilla and two on the lateral left dentary. The pathologies from the premaxilla are located at 45 cm from its anterior end (Fig 5b and 5c–5e), the one of the maxilla is at 75 cm from the anterior end of the premaxilla. The two lesions observed on the dentary are at 15 cm and 54 cm from the anterior end of the dentary (Fig 5b and 5f). The pathologies are aligned along an anterodorsal to posteroventral axis.

#### Ribs

Broken and healed ribs were described in the rib cage of SMNS 15950 by [10], supplemented by new observations [9]. Ribs 11–24, 30, 35 and 37–39 show callus development (Fig 5g and 5h), some also with pseudoarthrosis formation ([9], Fig 2d and 2e).

#### *Temnodontosaurus trigonodon* (GPIT/RE/9395). Posidonienschiefer Formation, lower–middle Toarcian, Frittlingen, Germany

**Fig 6:**
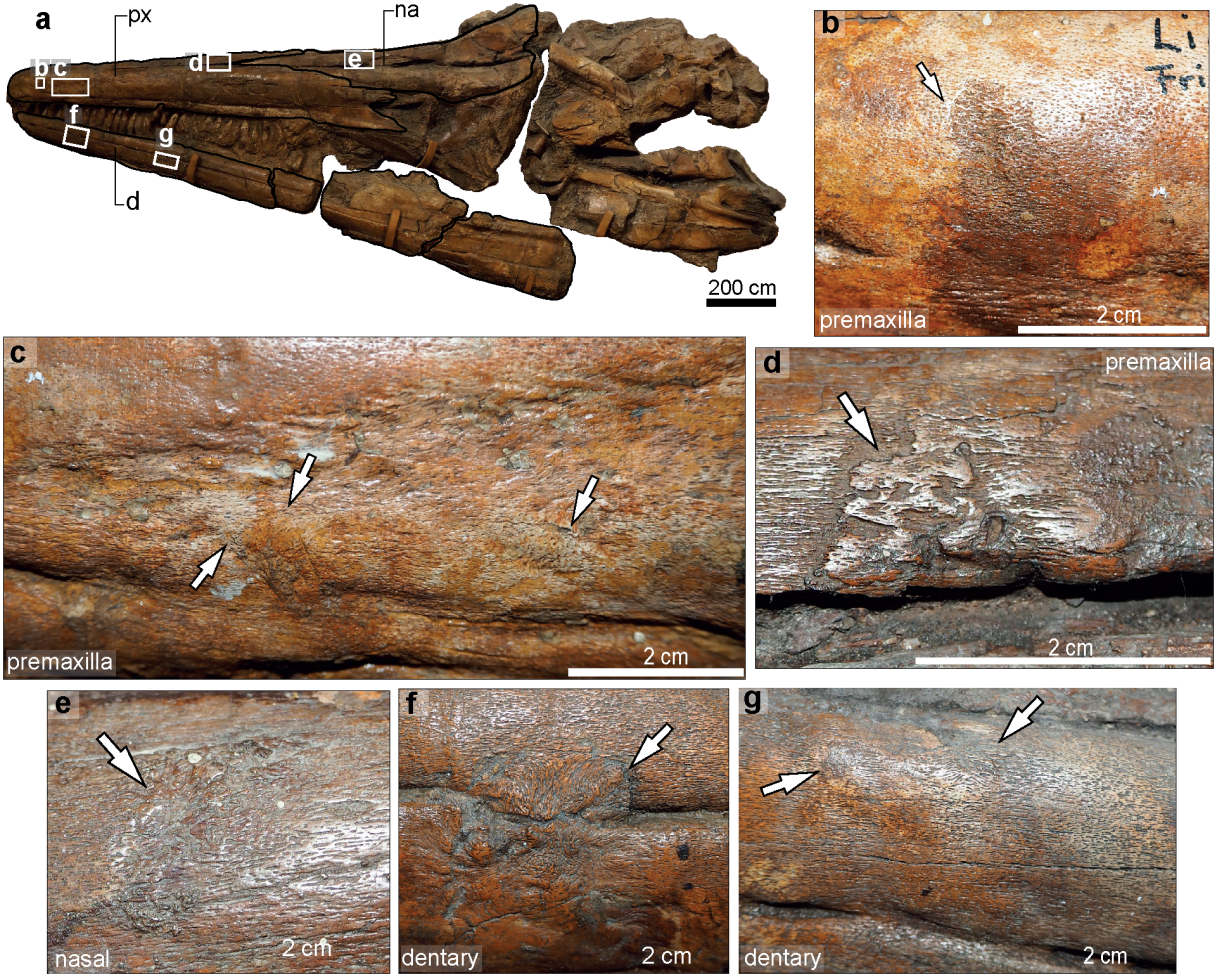
*Temnodontosaurus trigonodon* (GPIT/RE/9395). Posidonienschiefer Formation, ?lower Toarcian. **a**. Skull exposed in left lateral view showing pathologies on the premaxilla, nasal and dentary in b-g. **b**. Rugose area with fibre remodeling on the lateral surface of the premaxilla. **c**. Fibre remodeling on the ventrolateral margin of the premaxilla indicated with white arrows. **d**. Rugose-textured remodeled area on the posterodorsal surface of the premaxilla. **e**. Tissue remodeling on the dorsal surface of the nasal. **f-g**. Fibre remodeling on the lateral surface of the left dentary: **f**. at the level of the symphysis; **g**. at the dorsolateral margin.

An incomplete skull exposed in left lateral view (Fig 6).

The skull shows several areas with callus formation on the premaxilla, nasal and dentary (Fig 6b–6g). Three lesions are observed on the left premaxilla. The anteriormost, located on the anteriormost preserved premaxilla, is small and shows fibre remodeling (Fig 6b). The second is a large lesion, 73 cm from the external narial opening (Fig 6c). The third is at the anterior margin of the nasal (Fig 6d). A small lesion is located on the dorsal margin of the nasal, 38.5 cm from the anteriormost nasal (Fig 6e). The lesions are characterized by fibre remodeling and rugose texture. There are two areas with fibre remodeling on the left dentary. The first is located on the lateral dentary at the level of the dental groove, 127 cm from the posterior end of the dentary (Fig 6f). The healed area is roughly concentric in shape and shows callus development. The second, smaller lesion is located 20 cm posterior to the first (Fig 6g). It is located on the dentary ventral to the dental groove.

#### Non pathological bone modifications

Erosion of the bone surface caused possibly during seafloor exposure and enhanced during mechanical preparation was observed in the ventrolateral margin of the left dentary (GPIT/RE/9395; Fig 1b).

#### *Temnodontosaurus* sp. (UMH 1). Posidonienschiefer Formation, middle Toarcian, Holzmaden, Germany

**Fig 7:**
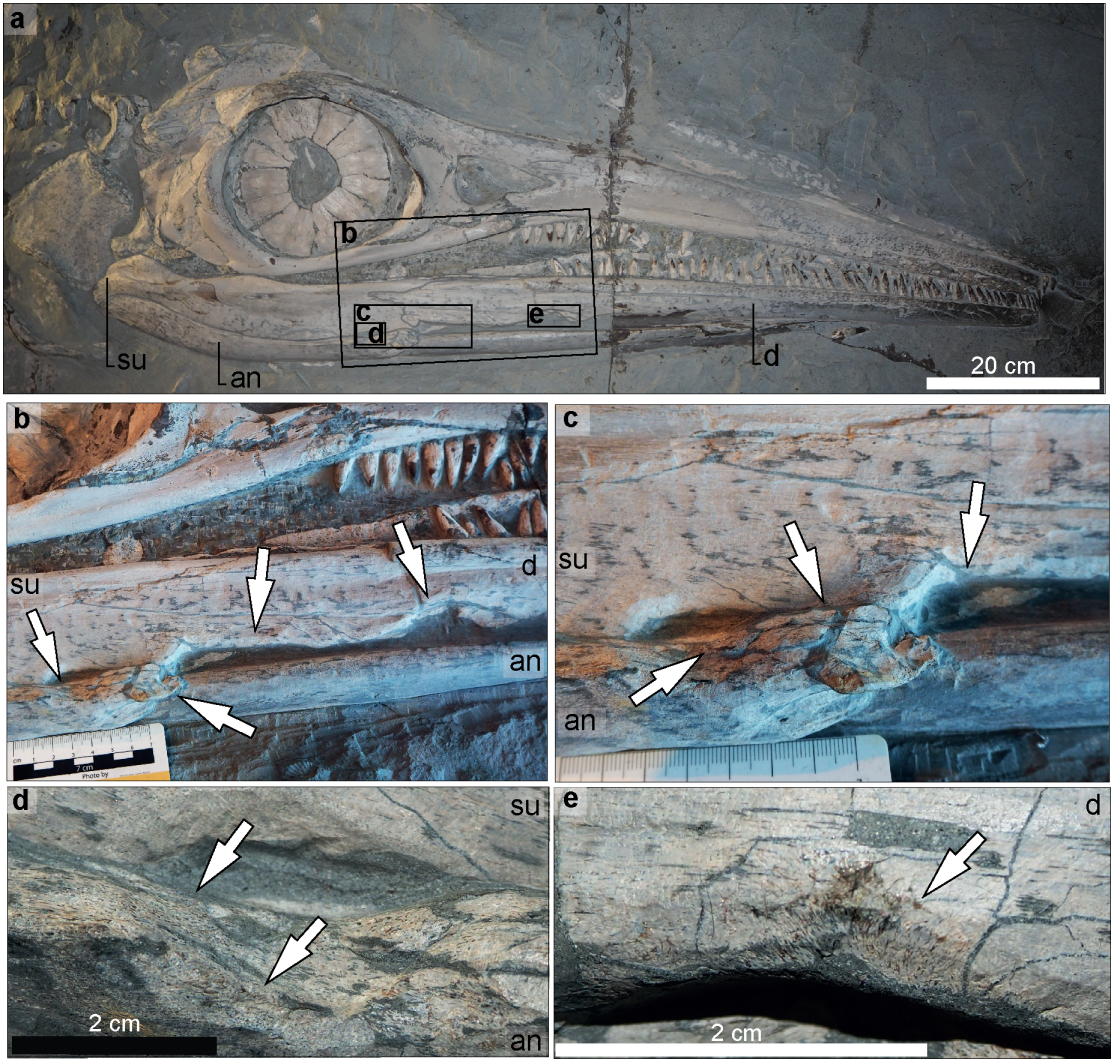
*Temnodontosaurus* sp. (UMH). Posidonienschiefer Formation, middle Toarcian. **a**. Skull in right lateral view indicating the pathological areas of the lower jaw illustrated in b-e. **b**. Concavities observed at the ventrolateral margin of the dentary and surangular. **c**. Bony overgrowth at the anterior end of the right angular. **d**. Tissue remodeling observed at the dorsal margin of the angular. **e**. Fibre remodeling on the lateral surface of the dentary, dorsal to the concavity.

An articulated skeleton, preserved in two dimensions and exposed in right lateral view (Fig 7).

#### Skull

Pathologies are present on the right mandible (Fig 7b–7e). On the ventral edge of the right dentary, a deep anteroposteriorly elongate concavity with remodeling along its edges is located 6.8 cm from the posterior-most end of the dentary (Fig 7b and 7e). A deep concavity is present on the ventral surface of the anterior surangular and ventral dentary (Fig 7b and 7c). Slight fibre remodeling and reactive bone are observed along the dorsal edge of the concavity on the surangular (Fig 7d). The anterior end of the right angular is deformed, as indicated by osseous overgrowth and an anteroposteriorly oriented groove between the surangular and angular (Fig 7c). Because of preparation, limited fibre remodeling is visible on the deformed sections.

#### *Temnodontosaurus trigonodon*. (GPIT/RE/1491/13). Posidonienschiefer Formation, lower Toarcian, Schlierbach, Germany

**Fig 8:**
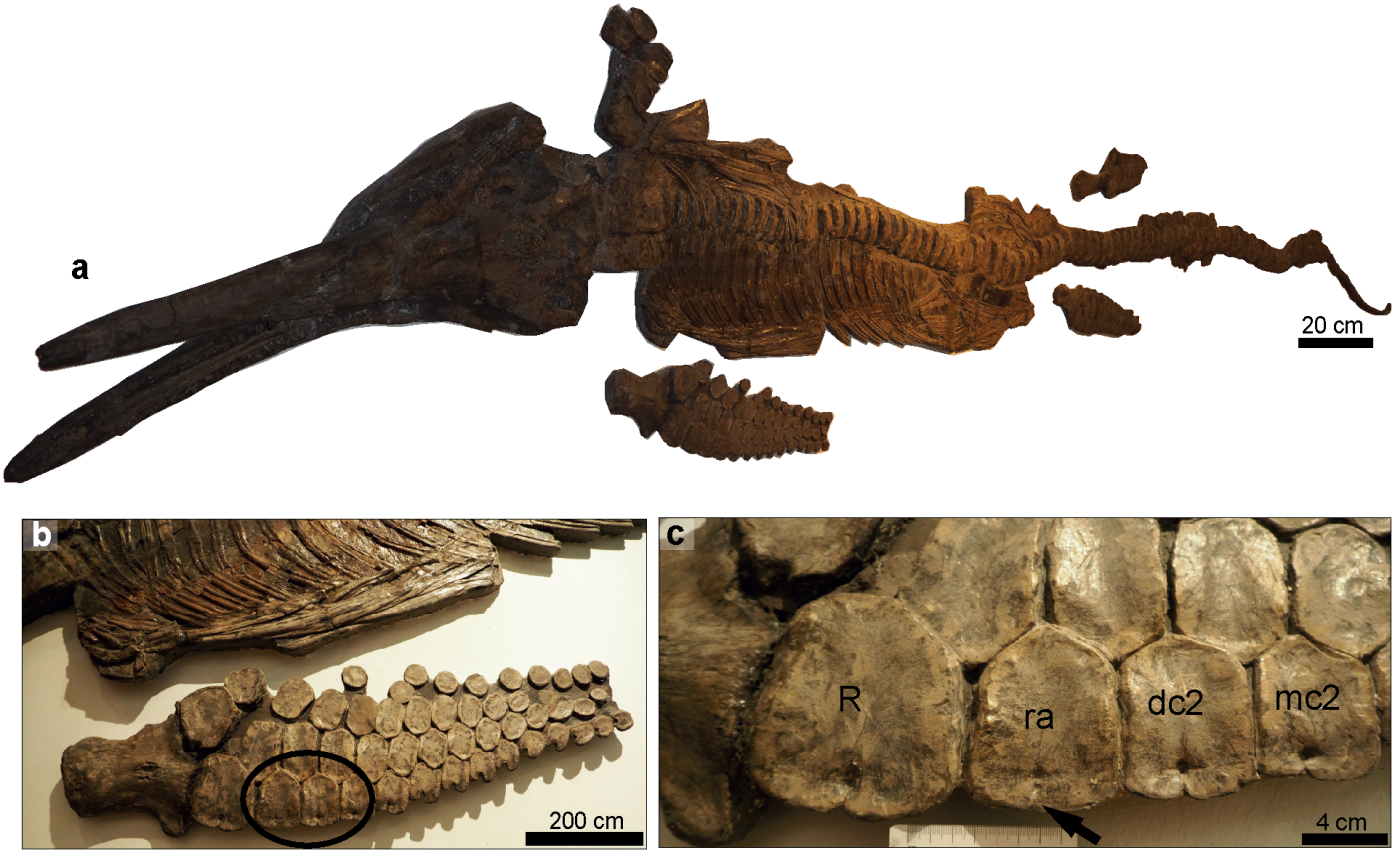
*Temnodontosaurus trigonodon*. (GPIT/RE/1491/13). Posidonienschiefer Formation, lower Toarcian. **a**. Complete skeleton in dorsal (skull) and ventral view (postcranium). **b**. Left forefin indicating the pathological elements in the black circle. **c**. Arrow indicating the complete occlusion of the anterior notch of the radiale. Partial occlusion is also observed in the anterior notch of the radius, distal carpal two and metarcarpal two. Abbreviations: **R**: radius. **ra**: radiale. **dc2**: distal carpal two. **mc2**: metacarpal two.

A complete and articulated specimen exposed in dorsolateral view (Fig 8a). The specimen shows pathologies in the left forefin: complete bony occlusion of the anterior notch of the radiale and partial occlusion of the anterior notch of the radius, distal carpal II, and metacarpal II (Fig 8b and 8c). There are no signs of fibre remodeling or callus formation but this specimen is severely polished by mechanical preparation, therefore any signs of healing might have been eroded during preparation. Occlusion of the anterior notches of forelimb elements is not within the range of normal morphology seen in Early Jurassic ichthyosaurs, and thus we consider this to be pathological.

#### *Temnodontosaurus trigonodon*. Jurensismergel Formation, upper Toarcian, Mistelgau, Germany, UM-O no. 4

An incomplete anterior skull with some postcranial elements, preserved in three dimensions ([14] pl. 1, Figs 1 and 2).

#### Skull

**Fig 9:**
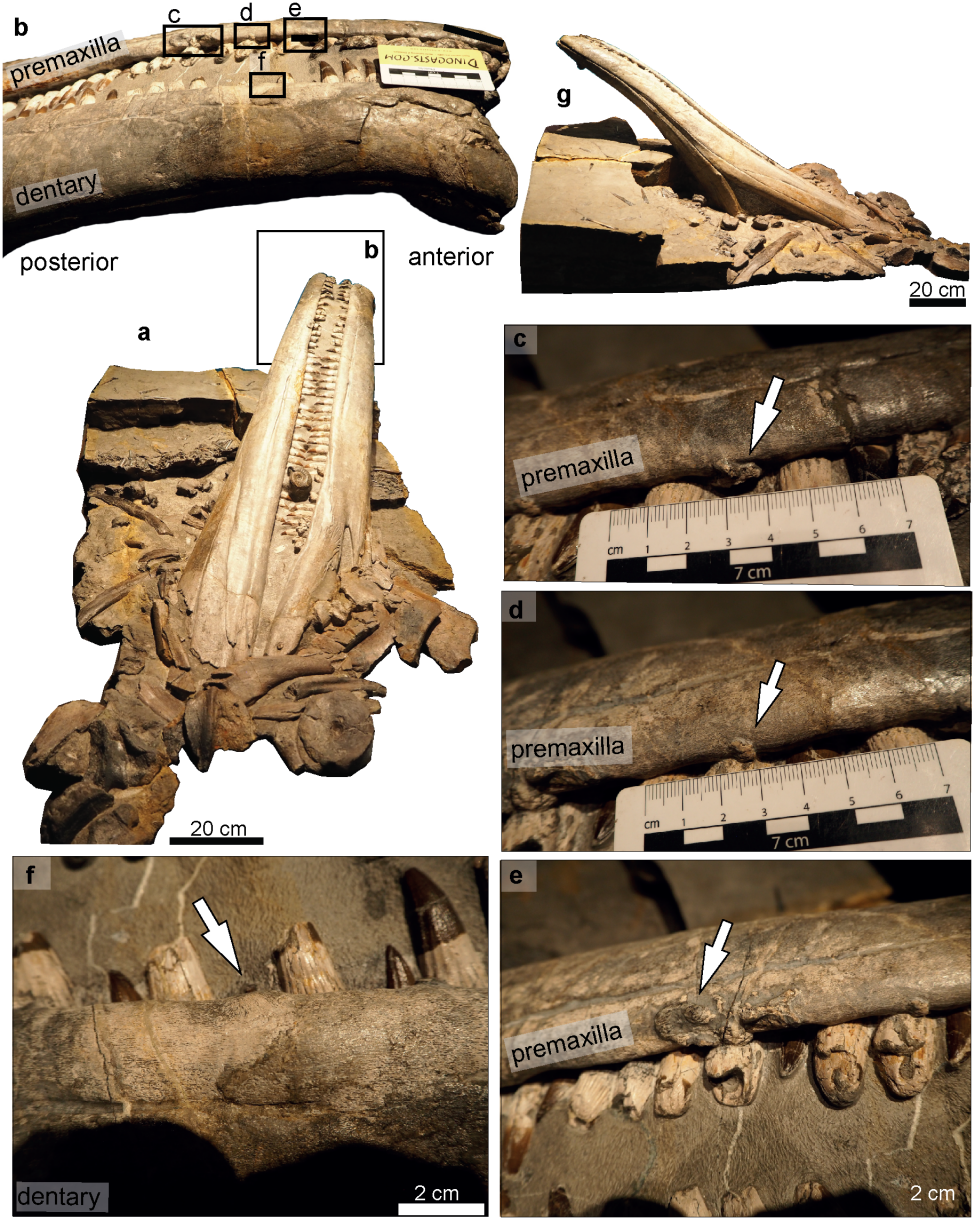
*Temnodontosaurus trigonodon*, Jurensismergel Formation, upper Toarcian. Rabold & Eggmaier (2013) U-MO specimen no. 4. **a**. Right lateral view of the skull; **b**. Anteriormost part of the premaxilla and dentary indicating the pathological areas in the right lateral premaxilla and dentary in c, d, e and f. **c-e**. Irregular tissue overgrowth at the ventral margin of the premaxilla. **f**. Tissue overgrowth with fibre remodeling at the dorsal margin of the dentary.

Pathologies are observed on the right premaxilla and dentary (Fig 9). Three areas with tissue overgrowth are observed along the lateroventral premaxillary margin (5.5 cm; 1 cm and 0.5 cm in length) at 20 cm from the premaxilla anterior tip (Fig 9a and 9b–9e). Fibre remodeling and reactive osseous tissue are present on the dorsolateral right dentary (Fig 9a and 9f). The pathology is located 12 cm from the anterior-most tip of the dentary and is approximately 3 cm in length.

#### *Temnodontosaurus trigonodon* (UMH 454). Posidonienschiefer Formation, lower–middle Toarcian, Holzmaden, Germany

**Fig 10:**
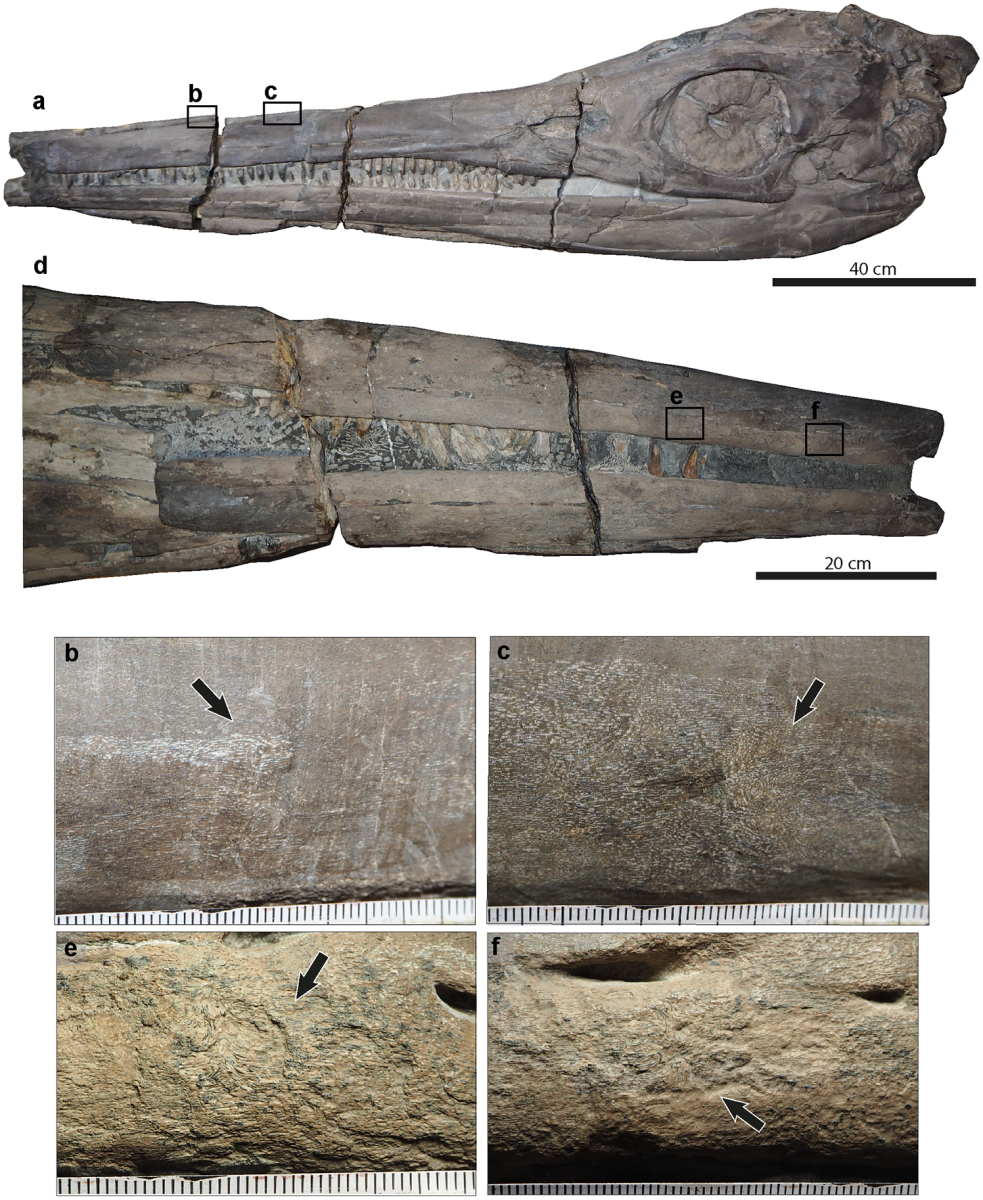
*Temnodontosaurus* sp. (UMH 454) Posidonienschiefer Formation. **a**. Skull exposed in left lateral view indicating the pathological areas in b and c. **d**. Right lateral view of the premaxilla indicating the pathological areas in e and f. **b-c**. Two areas with fibre remodeling at the dorsal margin of the left premaxilla. **e-f**. Two areas with fibre remodeling at the right lateral surface of the premaxilla.

A complete three-dimensional skull with a portion of anterior vertebral column and anterior pectoral girdle and forefins. Pathologies are observed in the left and right premaxillae (Fig 10). Two small areas with fibre remodeling are observed at the dorsal margin of the left premaxilla at 41 cm from the anterior tip (Fig 10a and 10b; Table 2). The second is located at 47 cm from the anterior tip of the left premaxilla (Fig 10a and 10c). The right lateral surface of the premaxilla shows two small areas with fibre remodeling at 13 cm and 24.5 cm from the anteriormost tip respectively (Fig 10d, 10e and 10f; Table 2).

**Table 2:** Measurements in cm of right and left pathologies observed in the premaxilla of UMH 454 (*Temnodontosaurus trigonodon*).

	Height	Length
Left premaxilla 1	1.2	1.4
Left premaxilla 2	3.1	3
Right premaxilla 1	2	2.5
Right premaxilla 2	2	2.2

### Frequency of pathologies in *Temnodontosaurus*

Among the *Temnodontosaurus* material surveyed, the skull is the most frequently represented anatomical unit in museum collections (34% of all specimens; Table 3; S1 Fig). The next most abundant anatomical units are the pectoral girdle and forefin (20%), followed by ribs (15%), pelvic girdle and hind fin (11%), and the anterior vertebral column (11%). The least frequently preserved anatomical unit of *Temnodontosaurus* in the collections is the posterior vertebral column (10%). These units appear to seldom be either preserved, collected, or prepared. More than 50% of the skeleton is represented in 18% (7/39) of the *Temnodontosaurus* specimens surveyed, whereas in 68% of the specimens less than 15% of the skeleton is preserved.

**Table 3:** Frequency of preserved anatomical units of *Temnodontosaurus*.

Anatomical module	Count	%
Skull	31	34%
Anterior vertebral column	10	11%
Posterior vertebral column	9	10%
Dorsal ribs	14	15%
Pectoral girdle and forefin	18	20%
Pelvic girdle and hind fins	10	11%
Total	92	100%

**Fig 11:**
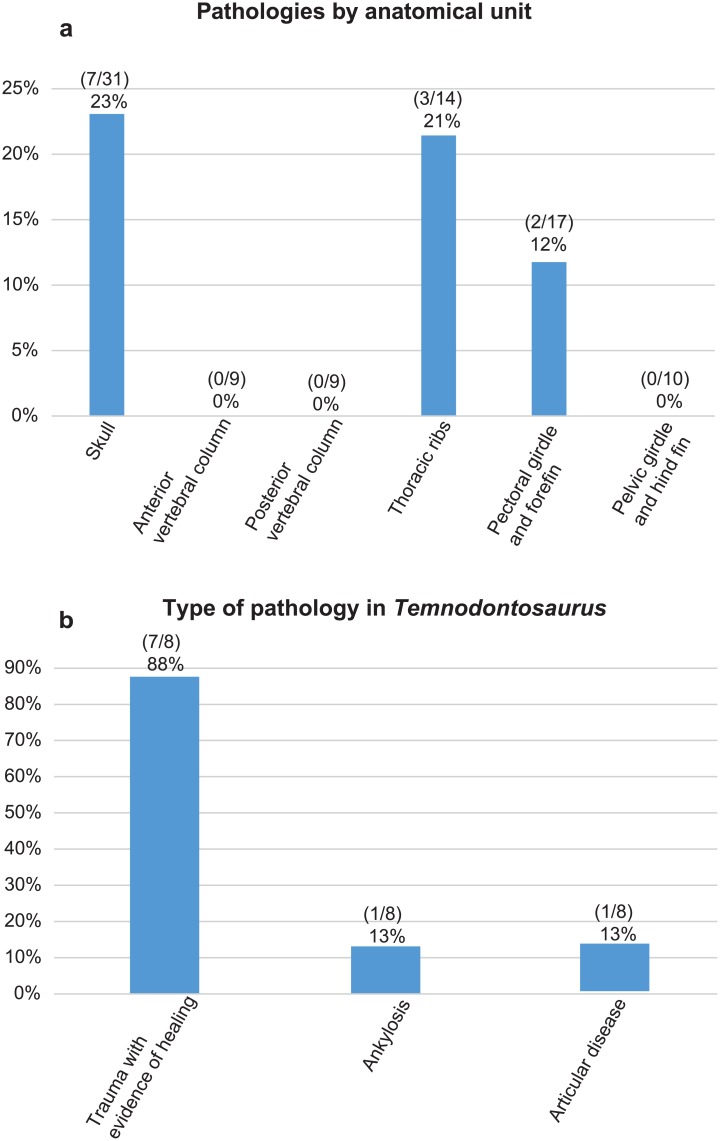
Graphic showing the relative frequency of pathologies by anatomical unit (a) and frequency of the types of pathologies observed in *Temnodontosaurus* (b).

Of the 39 specimens of *Temnodontosaurus* specimens analyzed, 8 (21%) showed osteological pathologies. Pathological specimens often displayed multiple pathologies per individual, when different anatomical regions are well-preserved. Pathologies were observed in three of the six demarcated anatomical regions (Table 4). The skull is the relatively most pathological anatomical unit (23% of all skulls (7/31), and 50% of pathological modules), followed by the dorsal ribs (3/14; 21%), and pectoral girdle + forefin (2/18 (11%); Fig 11a). Interestingly, the three cases with rib pathologies are all associated with cranial pathologies. No pathologies were observed in the vertebral column or the pelvic girdle and hind fin.

**Table 4:** Relative anatomical distribution of observed pathologies in *Temnodontosaurus*.

Anatomical region	N° of pathologies by anatomical unit	Total preserved by anatomical unit	%
Skull	7	31	23
Anterior vertebral column	0	10	0
Posterior vertebral column	0	9	0
Dorsal ribs	3	14	21
Pectoral girdle and forefin	2	18	11
Pelvic girdle and hind fin	0	10	0

Simple trauma with evidence of healing was the most frequently observed class of pathology in *Temnodontosaurus*, observed in 7 of the 8 pathological individuals (88%; Table 5; Fig 11b) followed by ankylosis (13%; 1/8). Articular disease was detected in only one specimen of *Temnodontosaurus* within our sample (U-MO [14] no. 14).

**Table 5:** Classification of pathologies observed in *Temnodontosaurus*.

Pathology	N°	Total pathological specimens	%
Trauma with evidence of healing	8	7	88%
Ankylosis	8	1	13%
Articular disease	8	1	13%

## Discussion

### Frequency and distribution of pathologies

Our systematic survey of pathologies within *Temnodontosaurus* has yielded a large number of previously unreported instances of skeletal damage, suggesting that extensive reporting bias is present in the primary literature. Previously, only broken ribs were noted in the specimens examined [10]; however cranial pathologies appear to be at least equally frequent. Among pathological specimens, both cranial and ribcage pathologies are more frequent in *Temnodontosaurus* than the reported ichthyosaurian average (50% and 25% of pathological modules vs. 10% and 15% of ichthyosaurian literature reports: [9]). This comes at the expense forelimb pathologies, which are relatively less frequently observed in *Temnodontosaurus* than reported in ichthyosaurs as a group. This was not unexpected since forelimb pathologies are more frequently reported in smaller-bodied taxa, correlated with increased incidence of articular disease [9]. As with other ichthyosaurian taxa, vertebral column and hind limb pathologies are consistently low or completely absent in *Temnodontosaurus*.

### Interpretation of the cranial and thoracic pathologies in *Temnodontosaurus*

The skull of UM-O no. 4 (Fig 9) shows reactive bone deposition along the labial edge of the premaxilla and dentary. Similar pathologies have been observed in the plesiosaurian *Pliosaurus* [17] and the extinct odontocete *Acrophyseter* sp. [18]. These have been variably interpreted as buccal exostoses formed as a result of occlusal stress, or as a consequence of malocclusion between the upper and lower jaws [17,18]. The location of the reactive bone lateral to the alveolar groove in UM-O no. 4 suggests jaw misalignment is the more likely cause of the pathologies in this individual. In at least one case (UM-O no. 14), the initial trauma to the lower jaw was followed by infection, leading to the formation of a large abscess. The pathological elements of the right pectoral girdle of the same specimen are most likely also due to an infection propagated following trauma. However, the right clavicle of this specimen was morphologically normal, suggesting that the infection in the lower jaw was not propagated posteriorly, but that there were two distinct points of infection. It is impossible to say with certainty whether the injuries to the ribs in any of the specimens were inflicted at the same time as the injuries to the jaws, however the coincidence of the cranial and rib-cage injuries in all three cases (UM-O 14, SMNS 15950, SMNS 13488) is noteworthy.

The systematic survey of osteological pathologies in *Temnodontosaurus* attempted here supports earlier observations that jaw infection and abscesses propagated through the pulp cavities of broken or heavily worn teeth are rare to absent in ichthyosaurs [9]. This type of jaw pathology is otherwise well-documented in cetaceans [19–21]. Polyphyodont tooth replacement in *Temnodontosaurus* might have played a role in limiting this type of mandibular infection and although dental caries have been reported in ichthyosaurs [22], continuous (polyphyodont) tooth replacement likely shed damaged or infected teeth before any bacterial infection could be transmitted to the jaw bone.

The dimensions and distribution of cranial injuries on the *Temnodontosaurus* specimens SMNS 13488 and SMNS 15950 (Figs 4 and 5), GPIT/RE/9395 (Fig 6), UMH1 (Fig 7), and UM-O no. 14 (Fig 3), prompt interpretation as healed traumatic injuries, possibly inflicted by another large marine reptile. The injury to the upper jaw in SMNS 15950 includes ten roughly circular areas separated by a few centimeters. The organization of the ten healed areas suggests more conclusively that this specimen sustained a bite on the rostrum from another large marine reptile. The size and spacing of the injuries are consistent with either a conspecific or a large-bodied coeval marine reptile, such as the crocodylomorph *Steneosaurus*. UM-O no. 14 (Fig 3) and SMNS 13488 (Fig 4) also suffered deep wounds to the posterior-most lower jaw. Both of these specimens also show healed injuries on the ventral splenial in the region of the mandibular symphysis. Such injuries are consistent with damage caused by aggressive encounters with other marine reptiles, or deep cuts inflicted by the mega-onychites of belemnitid coleoids, known as gastric contents in *Temnodontosaurus trigonodon* [6]. In general, the abundance of traumatic injuries to the lower jaw and rostrum imply that the soft tissue covering this region was relatively thin in *Temnodontosaurus*, since both *Steneosaurus* and *Temnodontosaurus* have tooth crowns only approximately 30 mm in height in large individuals, and Early Jurassic mega-onychites also fall into this size range.

Amongst other ichthyosaur taxa, healed bite marks attributed to interaction with conspecifics have also been documented in the Early Cretaceous taxon *Platypterygius australis* [23]. Recent analysis classifies *P*. *australis* as a macrophagous predator [24] on smaller-bodied vertebrates [25], with tooth crowns similar in size to those of *Temnodontosaurus* [26]. Non-fatal face biting behavior, (incurred either accidentally during feeding or as part of more complex social interactions), may have been widespread among ichthyosaurs. Indeed, a comparable injury recorded on the ventral angular of a specimen referred to *Leptonectes* cf. *tenuirostris* is also interpreted as a healed bite mark [27], although, the characteristically slender tooth and jaw morphology of this taxon (e.g., [26]), makes unlikely to have been inflicted by a conspecific.

Fractured ribs in ichthyosaurs have also been attributed to aggressive interactions with conspecifics [28], but collision with or beaching on reefs [29,30], or compression of the ribcage during deep diving [31] have also been proposed. The absence of avascular necrosis, also thought to be correlated with deep diving behavior (e.g., [11,32]), in any of the material reviewed during this study leads us to reject the hypothesis of [31]. The consistent association between broken ribs and facial traumas in *Temnodontosaurus* when both anatomical regions are adequately preserved strongly suggests that aggressive interactions, either with conspecifics or with other large contemporaneous marine reptiles, may be responsible for both types of skeletal pathology. As an aside, it should be noted that there is no evidence that injuries to the rib cage and skull were sustained at the same time–it is also possible that pain or reduced mobility caused by damage to the rib cage led to higher-risk feeding behavior and thus a greater chance for further injury during conflict with coeval predators or prey. Alternatively, aggressive mating behavior might also have contributed to traumatic pathologies, however, this is difficult to confirm in *Temnodontosaurus* given the ambiguities in determining sexual dimorphism.

Aggressive interactions with conspecifics causing significant skeletal damage are relatively common in apex predators. For instance, cranio-mandibular pathologies have been interpreted as caused by intraspecific combat, especially non-lethal biting behavior, in several theropod dinosaur taxa [33–35]. Injuries to the skull and jaws have likewise been described in both extinct and extant cetaceans. These have been hypothesized to be caused by intraspecific aggression [36,37] or interspecific aggression [38]. Evidence of trauma on the teeth and lower jaw has been hypothesized to be associated with intraspecific aggression in the extant odontocete *Physeter* [39]. Mandibular fractures in the short-finned pilot whale *Globicephala macrorhynchus* have been attributed as caused by intraspecific aggression and represented majorly in males than females [37]. However, it should be noted that in cetaceans bite marks resulting in osseous pathologies have never been reported. Other secondarily marine apex predators, such as mosasaurid squamates, show a range of osseous pathologies to the jaws; however these tend to be more severe than those observed in *Temnodontosaurus*. Additionally, and in contrast to cetaceans, fatal bite marks attributed to conspecifics have also been reported in mosasaurs [40]. [41] suggested head biting in the skull of *Tylosaurus nepaeolicus* according to healed punctures. [42] also described partially healed bite marks in the left dentary and left splenial of *Mosasaurus conodon* as a non-fatal attack by a member of the same species. Mandibular fractures are documented in several mosasaur taxa [43,44]. A severe infection of the posterior lower jaw, comparable to that observed in UM-O no. 14, has also been reported in *Mosasaurus hoffmanni* [45], however the cause of the infection is unknown. Dental abscess in the mandible of a Late Cretaceous mosasaur from Belgium was described by [46].

#### Articular disease

This detailed study of an Early Jurassic macropredatory ichthyosaur supports preliminary conclusions derived from meta-analysis [9] that the low rate of osteopathology reported in the ichthyosaurian vertebral column compared to other secondarily aquatic amniotes such as mosasaurs and cetaceans is real, and not related to reporting bias. Several cases of pathologies related to infections in the vertebral column of cetaceans have been described [47,48]; vertebral column pathologies are also common in plesiosaurs and mosasaurs [7,8,40,49–52]. This difference in the distribution of pathologies may indicate functional differences between the axial skeleton of ichthyosaurs and these other taxa. Large intervertebral spaces have been reported in some ichthyosaurs (e.g., *Ophthalmosaurus*: [53]) and the relatively quantity of soft tissue between the centra may have protected the ichthyosaurian axial skeleton from articular disease.

To date, avascular necrosis has been described in only one isolated femur of *Temnodontosaurus*, from the ‘Lower Lias’ of England [11]. The absence of subsidence structures consistent with avascular necrosis in our survey of *Temnodontosaurus* from southwestern Germany may reflect either the difficulty of identifying such structures in slab-mounted specimens, or may be of palaeoecological significance. Avascular necrosis has been hypothesized to be driven by predation pressure in ichthyosaurs [32] so either (a) *Temnodontosaurus* as the apex predator engaged in less high-risk diving behavior than its prey, or (b) prey species in the Posidonia Shale Sea were concentrated in a relatively narrow zone near the sea surface, avoiding euxinic bottom waters and thus making deep dives unnecessary. The latter is supported by the absence of avascular necrosis in other ichthyosaurs from southern Germany compared with the high frequency of this disease in species from the Oxford Clay and ‘Lower Lias’ of England [32].

## Conclusions

We noted perimortem scratches possibly attributable to scavenging by sharks, discolorations caused by postmortem epibiont encrustation, depressions attributed to deformation and compression, and tool marks caused during excavation and preparation. However, we also note a wide range of traumatic injuries to the skeleton in *Temnodontosaurus*, concentrated in the skull, ribs and pectoral girdle/forefins. This detailed atlas and analysis of the types of pathologies observed in the Early Jurassic apex predator *Temnodontosaurus* will provide a guide with which to distinguish pathologies from peri- or postmortem skeletal damage.

Most observed pathologies in *Temnodontosaurus* are traumas with signs of healing, possibly caused by bites and impacts sustained during aggressive encounters with conspecifics. Broken ribs are associated in most cases with other traumas in the skeleton such as injuries in the skull, which support this interpretation. The cranial pathologies observed in *Temnodontosaurus* are similar to those reported in cetaceans and mosasaurs, which were interpreted as traces of fighting or aggressive encounters. However, *Temnodontosaurus* differs from these other taxa in the absence of pathologies in the vertebral column, which can indicate a functional differentiation of these apex predators. The absence of avascular necrosis in *Temnodontosaurus* from southwestern Germany may also have a functional significance regarding habitat use differences between southern Germany and England. A survey of the frequency of avascular necrosis in articulated British material prepared as slab mounts will be necessary to test the relative influence of preservation/preparation bias on this pattern.

## Supporting information

S1 Fig Frequency of preserved anatomical units of *Temnodontosaurus*. (TIF)

S1 Table Total specimens of *Temnodontosaurus* studied in the museum collections. (XLSX)

S2 Table Percentage of completeness of the specimens of *Temnodontosaurus* from the museum collections, according to its anatomical units. (XLSX)

S1 File 3D Photogrammetric reconstruction of SMNS 13488. (PLY)

S2 File 3D Photogrammetric reconstruction of SMNS 15950. (PLY)

S3 File 3D Photogrammetric reconstruction of SMNS 13488 showing the area with fibre remodeling at the right and left premaxillae and left dentary (Fig 4a and 4e). (PLY)

S4 File 3D Photogrammetric reconstruction of SMNS 13488 showing the pathological area at the right lower jaw (Fig 4h and 4k–4n). (PLY)

S5 File 3D Photogrammetric reconstruction of SMNS 15950 showing the pathological area in the skull at the left premaxilla, maxilla and dentary (Fig 5b and 5c–5f). (PLY)

S6 File Instructions for viewing the 3D files in MeshLab software. (DOCX)
