# Supplementary figures and images for: Pathological survey on Temnodontosaurus from the Early Jurassic of southern Germany
Source: PLoS One. 2018 Oct 24;13(10):e0204951. doi: 10.1371/journal.pone.0204951 (PMC6200200; doi:10.1371/journal.pone.0204951)

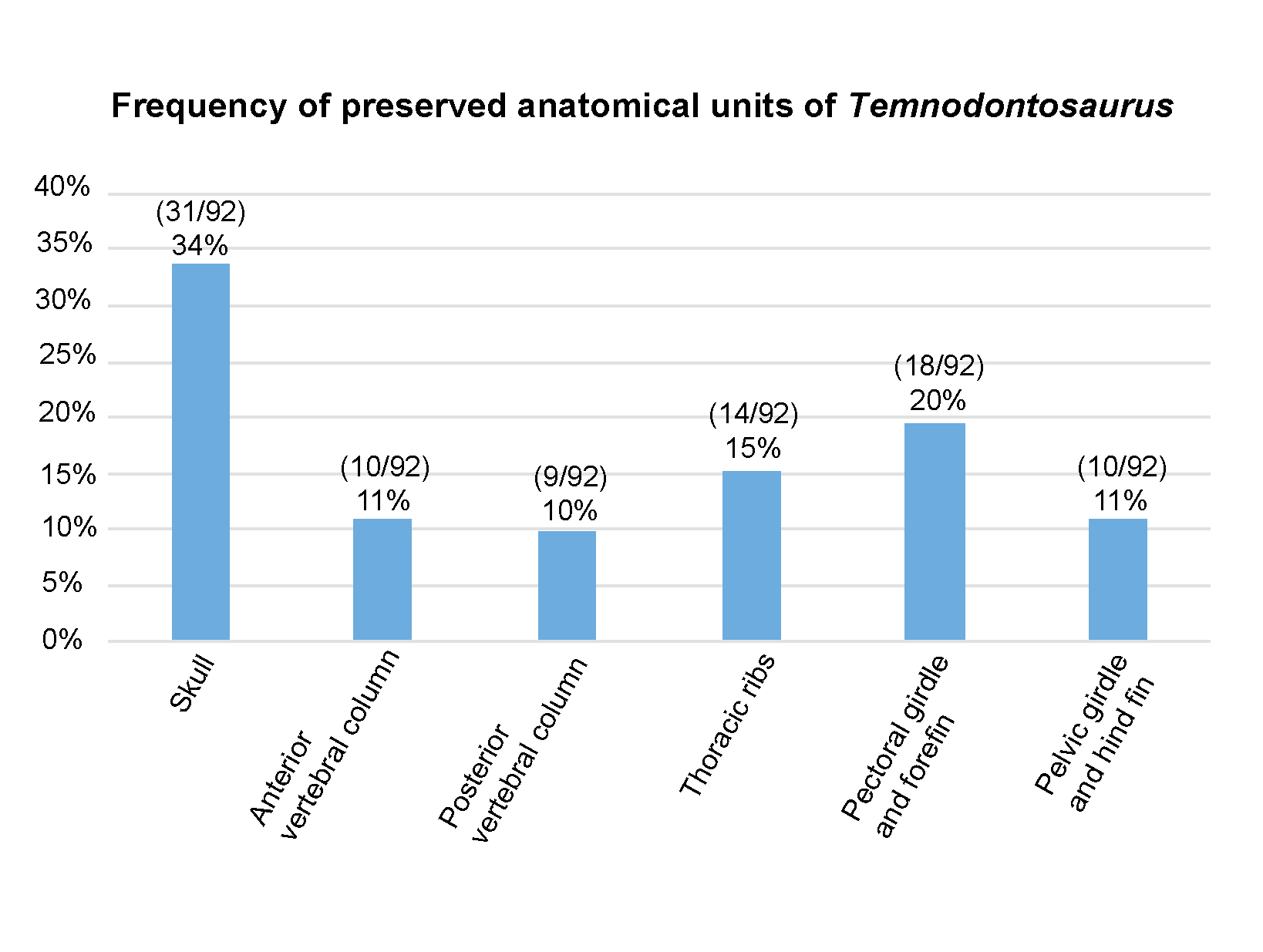

Supplement: S1 Fig — (TIF) [file pone.0204951.s001.tif]
