# Supplementary material for: Pathological survey on Temnodontosaurus from the Early Jurassic of southern Germany
Source: PLoS One. 2018 Oct 24;13(10):e0204951. doi: 10.1371/journal.pone.0204951 (PMC6200200; doi:10.1371/journal.pone.0204951)
Supplement: S6 File — (DOCX) [file pone.0204951.s009.docx]

**Instructions for viewing the 3D files in Meshlab**

MeshLab is an open source system for processing and editing 3D triangular meshes. The install file can be downloaded from <http://www.meshlab.net/#download>. Versions for Win 64, MacOS, and Linux are available.

In order to view a mesh, drag and drop it into Meshlab or open it via the *File* menu, option *Import mesh*.

Meshlab will automatically display the mesh in color. TO view the surface without color information, open the *Render* menu, select *Color* and choose *None* from the submenu. To turn colkor on again, select *per Vertex* in the same way.

The models can be rotated by clicking and holding the left mouse button and moving the mouse. The mouse wheel zooms in and out. Alternatively, zooming can be achieved by holding down the ALT key and clicking and holding the left mouse button. Moving the mouse up zooms out, moving it down zooms in. The model can be moved left, right, up and down my holding down the CTRL key, clicking and holding the left mouse button, and moving the mouse in the desired direction.

If several models are loaded into one Meshlab project window, a separate window opens automatically that shows all meshes in a list. Individual meshes can be hidden and shown by clicking the green eye icon to the left of the name in the list. This list window can be shown and hidden by pressing the CTRL and L keys, or via the *View menu*, *Show layer dialog*.
